# 

**Published:** 1922-10

**Authors:** 


### Ohio State Dental Society

### FIFTY-SEVENTH ANNUAL MEETING

### CINCINNATI, DECEMBER 5, 6, 7, 1922

President, C. H. Schott, 1004 Neave Bldg., Cincinnati
President-Elect, E. L. Pettibone, Cleveland
Vice-President, Harry Cope, Columbus
Secretary, F. R. Chapman, 305 Schultz Bldg., Columbus
Treasurer, A. O. Ross, Columbus

### CHAIRMEN OF COMMITTEES

**Chairmen of Committees—**
C. STANLEY SMITH, 511 Neave Bldg., Cincinnati, Ohio.
C. E. GERMANN, 717 Gwynne Bldg., Cincinnati, Ohio.

**General Arrangement—**
T. I. WAY, 52 Groton Bldg., Cincinnati, Ohio.

**Exhibits—**
W. E. GERMANN, 717 Gwynne Bldg., Cincinnati, Ohio.

**Legislation—**
H. C. BROWN, 609 Hartmen Bldg., Columbus, Ohio.

**Oral Hygiene—**
S. J. RAUH, 54 Harrison Bldg., Cincinnati, Ohio.
E. L. PETIBONE, 6503 Detroit Ave., Cleveland, Ohio.

**Membership—**
HARRY COPE, 328 E. State St., Columbus, Ohio.

**Publication—**
F. R. CHAPMAN, 305 Schultz Bldg., Columbus, Ohio.

**Board of Censors—**
J. F. STEPHAN, 1402 Guardian Bldg., Cleveland, Ohio.

The American Academy of Periodontists will hold its 1922 Meeting in conjunction with the Ohio State Meeting.

The "Register" will at a later date publish a list of the Essayists and Clinicians.

**Hotel Gibson, Fourth and Walnut Streets**

514 Rooms—All rooms with bath

| | RATES |
|---|---|
| Single room with bath | $2.50—$6.50 |
| Double room with bath | 4.25— 9.00 |
| Twin beds if desired | 5.00— 9.00 |

**Hotel Sinton, Fourth and Vine Streets**

750 Rooms—All rooms with bath

| | |
|---|---|
| Single room with bath | $3.00—$6.00 |
| Double room with bath | 5.50—10.00 |
| Twin beds if desired | 7.00—10.00 |

**Havlin Hotel, Vine and Opera Place**

192 Rooms — RATES

| | |
|---|---|
| Single rooms with bath | $3.00—$5.00 |
| Double rooms with bath | 5.00— 8.00 |

**Hotel Metropole, Sixth and Walnut Streets**

178 Rooms

| | |
|---|---|
| Single room without bath | $1.75—$2.25 |
| Single room with bath | 2.50— 3.50 |
| Double room with bath | 4.50— 7.00 |
| Double room without bath (men only) | 3.50— 4.00 |

**Grand Hotel, Fourth and Central Avenues**

255 Rooms

| | |
|---|---|
| Single room without bath | $1.50—$2.00 |
| Single room with bath | 2.50— 4.00 |
| Double room without bath | 2.50— 4.00 |
| Double room with bath | 4.00— 6.00 |

**Palace Hotel, Sixth and Vine Streets**

200 Rooms

| | |
|---|---|
| Single room without bath | $1.25—$1.50 |
| Single room with bath | 2.00— 3.00 |
| Double room without bath | 2.00— 3.00 |
| Double room with bath | 3.00— 4.00 |

**Emery Hotel 421 Vine Street**

150 Rooms

| | |
|---|---|
| Single room without bath | $1.50—$2.00 |
| Single room with bath | 2.50 |
| Double room without bath | 3.00— 4.00 |
| Double room with bath | 4.50 |

**Hotel Alms McMillan and Alms Place**

American Plan—150 Rooms

| | |
|---|---|
| Single room without bath | $3.00 |
| Single room with bath | 4.00 |
| Double room without bath | 6.00 |
| Double room with bath | 7.00 |

**Hotel Stag, 420 Vine Street**

90 Rooms

| | |
|---|---|
| Single room | $ .50—$ .75—$1.00 |
| Double room | .75— 1.25— |

Free use of shower and tub baths

**New Rand Hotel 25-27 W. Fifth Street**

115 Rooms

| | |
|---|---|
| Single room without bath | $1.00—$1.50 |
| Single room with bath | 1.50— 2.00 |
| Double room without bath | 1.50— 2.50 |
| Double room with bath | 3.00— 4.00 |

Free use of shower and tub baths

**Dennison Hotel, Fifth and Main Streets**

140 Rooms RATES

| | |
|---|---|
| Single room without bath | $1.00 and up |
| Single room with bath | 2.00 and up |
| Double room without bath | 3.00 and up |
| Double room with bath | 4.00 and up |

**Hotel Savoy (Stag), 5 E. Sixth Street**

90 Rooms

| | |
|---|---|
| Single room without bath | $1.50—$2.00 |
| Single room with bath | 2.50 |
| Double room without bath | 3.00— 4.00 |
| Double room with bath | 5.00 |

Use of shower bath

**Princeton Hotel, 431 Elm Street**

90 Rooms

| | |
|---|---|
| Single rooms | $1.00—$2.00 |
| Double rooms | 2.00— 3.50 |

Two baths on each floor, no extra charge

**Oxford Hotel, Sixth and Race Streets**

76 Rooms

| | |
|---|---|
| Single room | $ .75 per person |
| Double room | 1.00 per person |

Free use of shower and tub baths

---

**Cincinnati Hotel Rates Never Increased to Conventions**

We guarantee that if your meeting is held in Cincinnati there will be no increase in the regular rates of the hotels in this city; on the contrary if the members will advise us in time we will reserve our most desirable rooms for them, affording them our best accommodations without additional cost.

CINCINNATI HOTEL MEN'S ASSOCIATION
E. W. LYND, *President.*

---

### CINCINNATI—THE CONVENTION CITY

### *"The Athens of America"*

### A Picturesque Locality

Built on seven hills, Cincinnati is often called the Athens of America. Its suburbs impart a picturesque beauty to the city and the views from the hilltops are inspiring indeed. Go to Eden Park and see the historic Ohio winding between the Ohio and Kentucky shores on its course southwest to Louisville and beyond. It is a sight particularly worth while. Another wonderful view of the Ohio, of eastern Cincinnati, and of the four Kentucky cities —Covington, Newport, Bellevue and Dayton—is obtained from the observation pergola on Ingleside Place, Walnut Hills. Take a Gilbert avenue car and get off at Woodburn and McMillan.

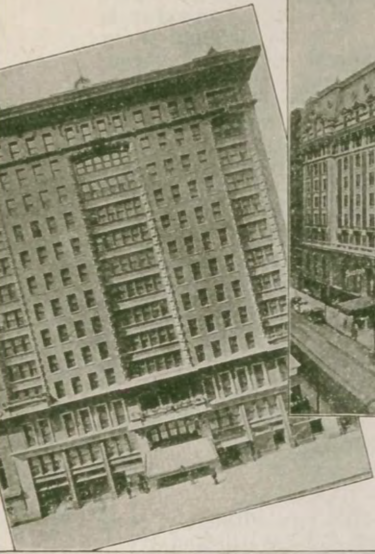

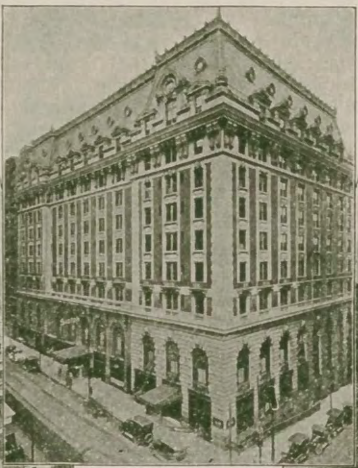

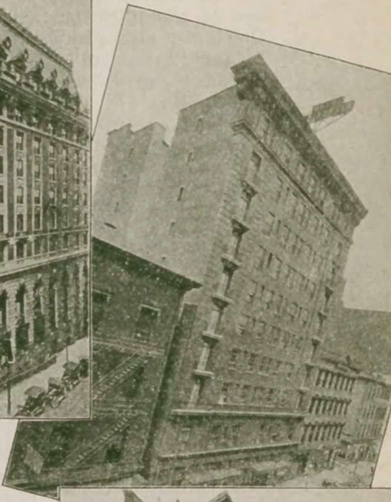

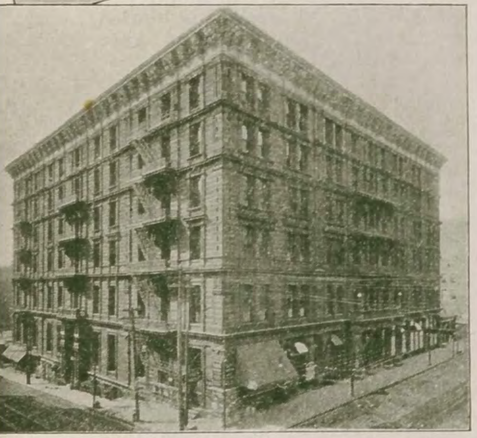

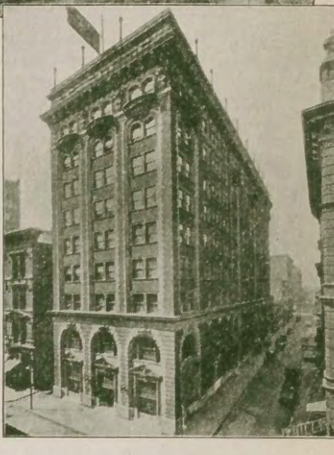

**Gibson Hotel** **Sinton Hotel** **Metropole Hotel**

**Grand Hotel** **Havlin Hotel**

Having obtained these glimpses of the topography of the Queen City, you may decide to visit the Art Academy in Eden Park—always a source of interest to visitors and citizens alike—and the Rookwood Pottery near-by in Mt. Adams, famed the world over for its ceramic masterpieces. Leaving the pottery you should visit the monastery and shrine on Mt. Adams where, once a year, pilgrims fervently implore the blessing of the Holy Virgin.

Clifton is not far away. There is located Hughes High School, one of the most beautiful specimens of high school architecture in the land; also Cincinnati University, the only municipal university in the States, and the birthplace of co-operative engineering education. Burnet Woods, an interesting natural park, adjoins the University.

Next you will want to see the Cincinnati General Hospital, municipally owned and one of the largest institutions of its kind. Go thence to the Zoological Gardens and revel in their scenic beauty. The Zoo embraces a collection of 2,000 birds, beasts and reptiles, some exceedingly rare.

### A Musical Center

If interested in music, you should visit the Cincinnati College of Music and Conservatory of Music, where many famous musicians have been developed. The Beecher homestead, on Gilbert avenue, where Harriet Beecher Stowe lived while she gathered material for "Uncle Tom's Cabin," is interesting to visitors. Also the old Garrett House, down-town, where Thomas Buchanan Read wrote "Sheridan's Ride."

### Parks and Amusements

There are many beautiful parks and residential sections, chief among them being Eden Park, Burnet Woods, Mt. Echo, Ault Park and Washington Park. Don't leave the city without visiting Walnut Hills, Clifton, Avondale, Hyde Park, College Hill and Westwood, six of our most beautiful suburbs. In College Hill is located the Ohio Military Institute and Clovernook, the home of Alice and Phoebe Cary, now an institute for the blind.

Amusement resorts are Coney Island, ten miles up the river; Chester Park, and the Zoo. In summer, concerts are given afternoon and evening at the Zoo and on Sunday at Eden Park and Burnet Woods.

A trip to the tower of the Union Central Building—the Woolworth Building of the Middle West—is another feature. You get a truly wonderful view of the City and the surrounding Kentucky hills. The Ohio Mechanics Institute has much to offer those interested in science and industry.

Car rides may be taken through the Kentucky hills on the Ft. Thomas and Ft. Mitchell cars. Rides through interesting sections of Cincinnati can be taken on the Zoo-Eden, Vine-Burnet, Madisonville, North Norwood, Clifton-Ludlow, and Vine-Clifton lines.

Cincinnati, the most southern northern city, and the most northern southern city, is located on the Ohio River midway between its source and its mouth. The business section on a level plateau about 125 feet above the river, is surrounded by picturesque hills which rise several hundred feet higher.

### Places and Things of Interest

**Art Academy**—Eden Park, close to Art Museum. Sent out many artists whose works have spread the fame of Cincinnati throughout Europe and America. Has students in drawing, painting and decorative art. Car No. 49.

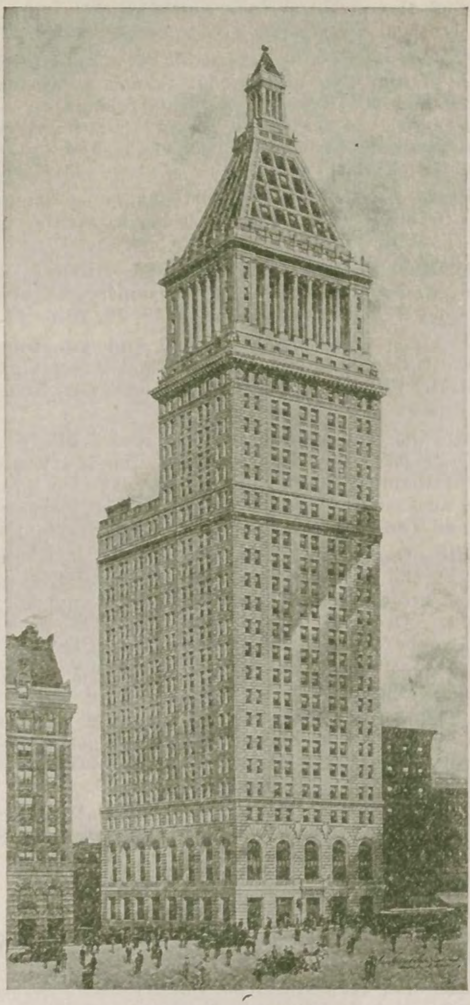

Union Central Bldg.

**Art Museum**—Eden Park. Dedicated 1886. Collection consists not only of sculpture and paintings, but is rich in textiles, ceramics, metal work, carvings, costumes, arms and armor, musical instruments, etc. Car No. 49.

**Beecher, Home of Lyman**—N. E. corner of Gilbert and Foraker avenues, Walnut Hills. Here Harriet Beecher Stowe lived while she was gathering material for "Uncle Tom's Cabin," and meeting the originals of the persons that figure in this story. Car Nos. 5, 7 or 9.

**Burnet Woods**—Adjoining grounds of Cincinnati University, directly north of and two miles from Fountain Square. Contains over 163 acres and is covered with grand old forest trees. Lake of about three acres. In the summer, every Saturday, band concerts are given. (Cincinnati University may be seen on the trip.) Car Nos. 55, 61 or 65.

**Chester Park**—In north-central part of city, summer amusement park, May to October. Numerous concessions, bathing and boating. Car Nos. 15, 41, 47 or 56.

**Cincinnati Baseball Park—Redland Field**—Findlay, Western and York avenues. One of the finest in the country. Seating capacity over 25,000. Cost $400,000. Car Nos. 15, 17, 18, 20 or 21.

**City Hall**—Constructed of granite and Amherst stone; cost over $2,000,000. The tower is 32 feet square and 250 feet high. Situated at Eighth, Ninth, Plum and Central avenue. Car Nos. 17, 21, 32, 33 or 34.

**Clovernook**—On the Hamilton Pike, near College Hill. This was the home of Alice and Phoebe Cary. The Cary property was purchased by William A. Procter and presented to the Trader Sisters, to be used as an institution for the blind. The historic homestead is now known as The Clovernook Home for the Blind. Car No. 17.

**College Hill**—One of Cincinnati's most picturesque suburbs. Contains one of the finest private residences in the country, the Thomson home, an exact reproduction of the French Palace, The Petete Trianon. Also the Ohio Military Institute, the homestead of Alice and Phoebe Cary. (See Clovernook.) Car No. 17.

**College of Music**—Adjoining Music Hall, Elm street, north of Twelfth. Is not conducted for profit. Its entire income is devoted to increasing its teaching facilities, and giving of numerous musical and dramatic events for the benefit of its students and the community at large and for the assistance of exceptional talent. Car Nos. 16 or 23.

**Coney Island**—On the Ohio River ten miles above city, Summer Amusement Park, open from June 1 to September 1. Reached by pleasure steamers leaving foot of Broadway. Delightful river ride.

**Conservatory of Music**—Highland, Burnet and Oak. Car Nos. 44 or 46.

**Consolidated Railroad Ticket Office**—Gwynne Building, Sixth and Main streets.

**Court House**—The New Hamilton County Court House at Canal and Main streets cost $2,500,000, and is one of the finest in the country. Car Nos. 43, 53, 61, 64, 76 or 78.

**Dixie Terminal Building**—S. W. corner Fourth and Walnut streets. Northern terminal for all green (Kentucky) cars. Has one of the finest arcades in the country.

**Eden Park**—One of the city's largest and most beautiful parks. Located in Walnut Hills on the crest of Mt. Adams. Commands fine views of the city and several of its suburbs, and the Ohio River for several miles. Contains lakes, reservoirs, greenhouses, a water-

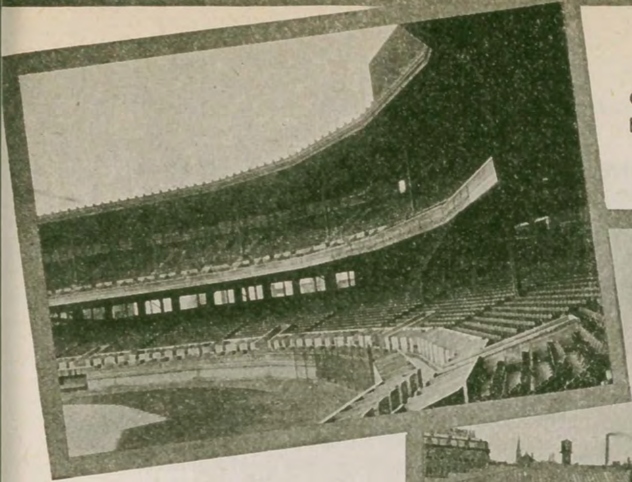

CINCINNATI'S NEW $400,000 BASEBALL GRANDSTAND.

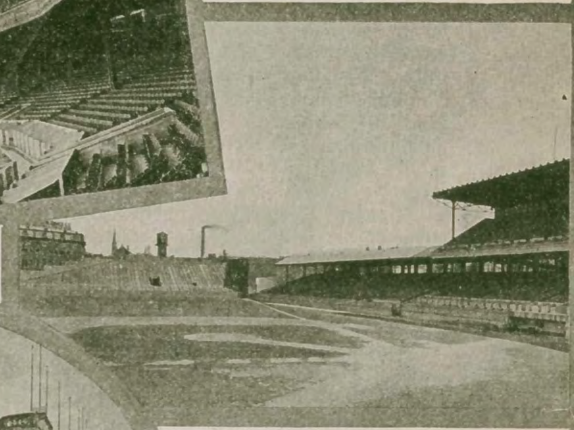

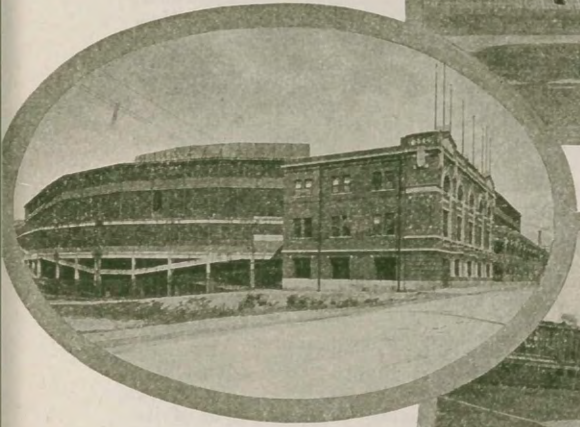

Cincinnati Had The First Baseball Team in America

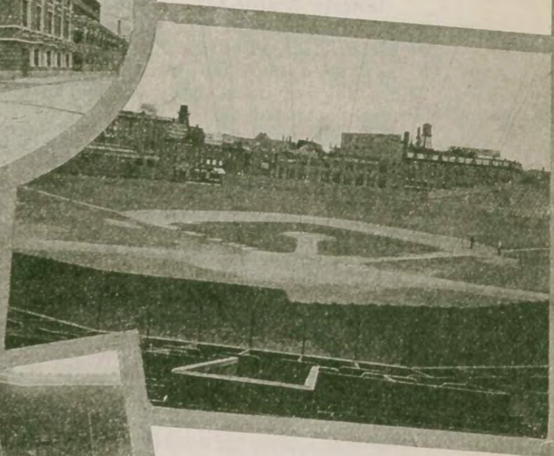

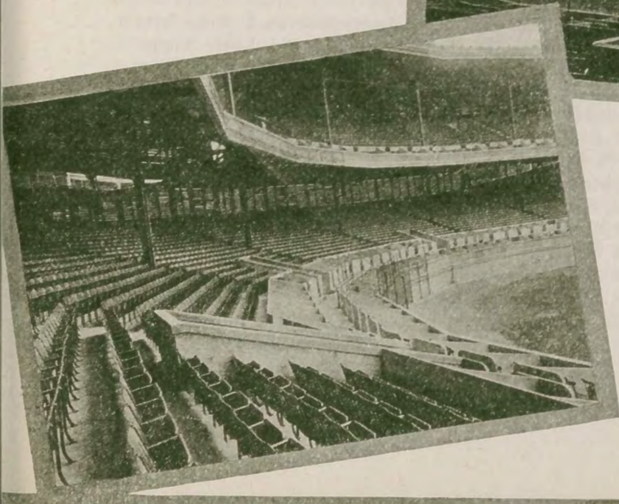

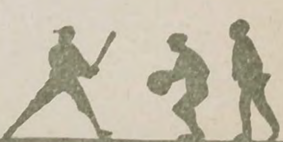

Cincinnati is Headquarters of the National Baseball Commission tower, beautiful driveways and stone-arched bridges, and an abundance of trees and grass, with rest and shelter houses. Area, 400 acres. Car No. 49 passes through the park. Car Nos. 2, 3, 68, 69 or 70 pass the Gilbert avenue entrance. Car No. 49 passes the Art Museum and Art Academy. Band concerts Sunday afternoons during the summer season.

**Fort Thomas**—A reservation of the United States, in the Highlands of Kentucky. Used as an infantry army post. Area, 111 acres, 59 buildings Water-tower, a beautiful stone structure at entrance, is 102 feet high. Magnificent view of Ohio River and Little Miami River valley. Fort Thomas (green) cars at Fifth and Walnut.

**General Hospital**—Fronting on Burnet avenue. Hospital grounds cover 65 acres. Thirty-eight acres are yet unimproved, and will later be used as a convalescent park. Has 24 buildings, 19 in the general group and 5 in the contagious group. Capacity, 1,300 patients. Cost, $4,000,000. Finest municipal hospital in the world. Car Nos. 44, 46 or 49.

**Government Building and Custom House**—A magnificent granite structure which cost over $6,000,000. It is located on the north side of Fifth, between Main and Walnut streets.

**Latonia Race Course**—Located in a suburb of Covington across the Ohio River on the Kentucky side. Latonia (green) cars on Fourth, between Walnut and Vine streets. (Time about 40 minutes.)

**Lincoln Statue**—Cincinnati's newest monument. In Lytle Park, near end of East Fourth street. Unveiled March 31, 1917. The gift of Mr. and Mrs. Charles P. Taft to the city of Cincinn[illegible]i. It depicts a man as a man more than any other public sta[illegible]ue in the city. The beauty of its artistry lies not in idealizing the subject, but in a correct portrayal of the man's physical characteristics as conceived by the artist, George Gray Barnard. (Walking distance.)

**Literary Club of Cincinnati**—25 East Eighth street. This was organized in 1849, and has been continuously identified with every literary activity of Cincinnati. It is probably the oldest club of its kind in the United States. Walking distance, or Car Nos. 7, 15 or 55.

**Lloyd Museum**—224 Court street, near Elm; free admission. Collection of dried fungi larger than in all other museums in the world combined. One-half block west of No. 303 West Court street is the Lloyd Library, with 20,000 volumes.

**Lytle Park**—On the square between Lawrence and Pike and Fourth and Third streets. Here is the site of the old Lytle homestead, built in 1810 by the grandfather of the poet and soldier, General William H, Lytle, author of the famous lyric "Anthony and Cleopatra." The old building was torn down in 1898, and the property converted into a public park. Here may be seen Barnard's statue of Lincoln. (See Lincoln Statue.) Walking distance.

**Music Hall**—Elm street, opposite Washington Park. Built in 1877. Has one of the finest pipe organs in the country. Size of hall, 112 feet wide and 192 feet long. Seating capacity, 3,600. Stage, 112 feet in width and 70 feet deep; one of the largest in United States. Car Nos. 60 or 63.

**Observatory, First Cincinnati**—On the summit of Mt. Adams. The cornerstone of this observatory, the first in the United States,

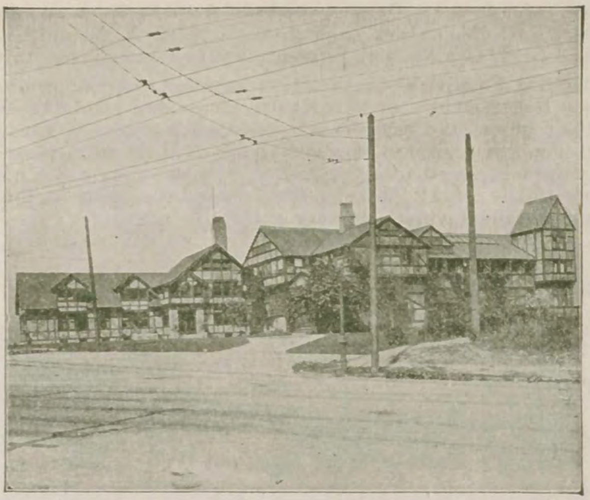

**Rookwood Pottery**

was laid by John Quincy Adams in 1843. The observatory was founded by General Ormsby M. Mitchell, and up to the time of the Civil War, it was the finest observatory in the United States. The building is now a monastery. Car No. 49.

**Observatory**—Of the University of Cincinnati at Mount Lookout, six miles northeast of the center of the city. Grounds comprise four acres on the summit of a hill. Contains very large telescope. Car Nos. 68 or 69.

**Ohio Mechanics Institute**—For nearly a century has been a factor in the industrial and scientific world. Building at Walnut, Canal and Clay streets is equipped to accommodate 4,000 students. Was built by a gift from Mary M. Emery of $600,000.

**Parks and Playgrounds**—Seventy-seven public parks, 2550 acres, 21 athletic fields with 32 baseball diamonds, 17 tennis courts; 24 equipped and supervised playgrounds with wading pools and shelter houses. Four golf courses. Extensive boulevard system partly completed.

**Public Library of Cincinnati**—Vine street between Sixth and Seventh streets. Contains 500,000 books. Has 125 newspapers and 800 magazines on file. Contains latest directories of 40 American cities and of London Paris and Berlin. Circulates sheet music, music rolls, stereopticon pictures, and has 20,000 lantern slides to circulate. Has 23 branches and 26 stations scattered over the city and county. Has 171 class rooms and school deposit libraries. Contains books in raised type for use of blind patrons. It sends these books to every State in the Union. Walking distance.

**Rookwood Pottery**—On the brow of Mt. Adams, overlooking downtown district. Known all over the world for exquisite specimens of ceramic art. Open to visitors 9 to 11:30 A. M. and 1:00 to 4:00 P. M. Saturdays from 9:00 to 11:00 A. M. only. Guides furnished free. Car No. 49. Fifteen minutes from downtown section.

**Shopping Facilities**—Cincinnati is an ideal shopping center. The immense Department stores, exclusive shops and specialty houses—with their offices and representatives in all markets of the world—assure shoppers a choice of merchandise that is unsurpassed from every viewpoint, particularly variety. Special sales, which are being held practically at all times, enable those who shop in Cincinnati to secure excellent values at very attractive prices.

### Theatres

GRAND........................Musical Plays, Comedies and Drama
Vine, between Fifth and Sixth

LYRIC........................................Motion Pictures
Vine, between Fifth and Sixth

KEITH'S..........................................Vaudeville
Walnut, between Fifth and Sixth

PALACE..............................Vaudeville, Motion Pictures
Sixth, between Vine and Walnut

SHUBERT'S....................................Musical Plays
Seventh and Walnut

GEO. B. COX MEMORIAL....................Comedy and Drama
Seventh, between Vine and Walnut

EMPRESS..........................................Burlesque
Vine, between Eighth and Ninth

OLYMPIC..........................................Burlesque
Seventh, between Walnut and Main

CAPITOL......................................Motion Pictures
Seventh and Vine

WALNUT.......................................Motion Pictures
Walnut, between Sixth and Seventh

STRAND.......................................Motion Pictures
Walnut, between Fifth and Sixth

FAMILY.......................................Motion Pictures
Vine, between Fifth and Sixth

EMERY AUDITORIUM......................Symphony Orchestra
Walnut and Canal

**The Cincinnati Symphony Orchestra,** conducted by Fritz Reiners, lately conducted by Eugene Ysaye, is a notable musical organization which you can hear while in Cincinnati.

**The Ohio College of Dental Surgery,** located at Seventh and Mound streets, is particularly interesting to all dentists as it is one of the early colleges which have helped hue the way for success in the Dental Profession.

**The Cincinnati College of Dental Surgery,** located at Court and Plum streets, also is particularly interesting and deserving a visit while in Cincinnati.

Cincinnati streets are numbered east and west from Vine street and north from the Ohio River.

**Tyler Davidson Fountain**—Fifth, between Vine and Walnut streets. Presented to the city by Henry Probasco. Designed by August Von Krehling, and cast by Ferdinand Von Muller, Trustee of the Royal Bronze Foundry of Bavaria. Unveiled in 1871. Cost over $200,000.

**The Union Central Life Building**—Fourth and Vine streets, 34 stories, 495 feet above, and 4 stories, 40 feet below the street level. The tallest building of any inland city in the world. The cost of the building and lot, $3,584,000. The home office of the Union Central Life Insurance Company, a Cincinnati institution. Guides furnished. Visitors welcome. It is also the home of the Cincinnati Chamber of Commerce.

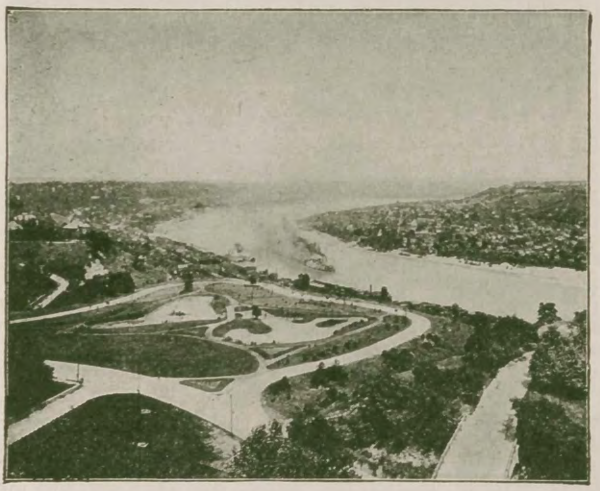

**View of Ohio River from Eden Park**

**University of Cincinnati**—Burnet Woods, comprises McMicken Hall, Cunningham Hall, Hanna Hall, Van Wormer Library, Engineering Building, Chemistry Building, Gymnasium, Power Plant, and the stadium of the athletic field. It covers about fifty acres at the southern end of Burnet Woods. It is the only municipal university in the United States, being supported by taxes of the city, and the entire group of buildings cost about $2,000,000. The Cincinnati University is further famous as the birthplace of the co-operative system of engineering education, in which young men work half time in the University and half time in the shops of the city, which feature has attracted the attention of educators all over the world and has brought to Cincinnati many ambitious young men who desire to obtain a superior training in engineering. The new Medical College of the University is situated a short distance from the University, on the grounds of the magnificent new city hospital, and cost over $500,000.

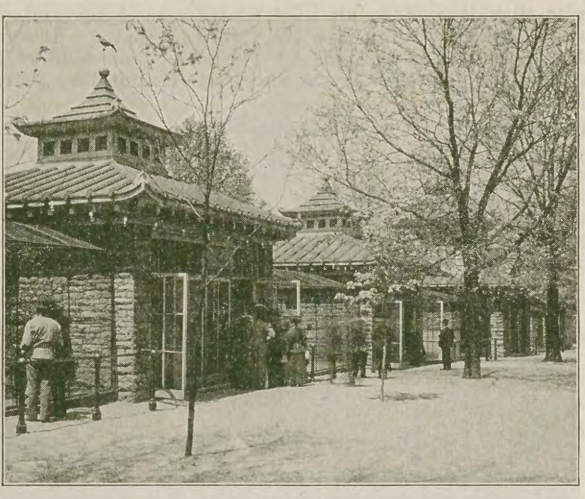

**Zoological Garden**

**Water Works**—Cincinnati now has one of the finest pumping and filtering plants in existence. Water is so pure as to be used direct from faucet, even in hospitals. Great engineers from all parts of the world have visited the plant at California, a Cincinnati suburb. Extreme eastern end of city, at a point considered one of the most picturesque on the Ohio River. Car Nos. 27 or 28.

**Zoological Garden**—North-central part of the city and directly at the head of Vine street. Area over 63 acres. Has one of the largest animal collections in the United States, and its reputation extends around the world, not only on this account, but also for its scenic beauty; fine clubhouse on the grounds; concerts twice daily throughout the summer season. Has 2000 birds, beasts and reptiles, finest giraffe in the world, biggest hippopotamus in the United States, largest buffalo. Grounds and animal houses open every day in the year. Car Nos. 49, 53, 54, 76 or 78.

### Noted for Art Institutions

Cincinnati's attitude toward art is a reflection of its love for music. It had the first endowed Art Academy in America. The Art Museum and Art Academy cost $330,000. Besides sculptures and paintings, the Museum collections are rich in textiles, ceramics, metal work, carvings, costumes, arms and armor, musical instruments, and like art productions. The Art Academy has 400 students in painting and decorative arts. Famous paintings and art treasures are housed in it and in several private collections.

The Art Academy proves the quality of its methods by supplying most of the decorators, both men and women, for the famous Rookwood Pottery. The pottery was founded in 1880 by Mrs.

Maria Longworth Storer and is managed on lines opposite to the prevailing factory system, the effort being made to attain higher art than cheaper processes. With clays entirely American and largely from the Ohio Valley, the Rookwood Pottery has achieved exquisite specimens of ceramic art.

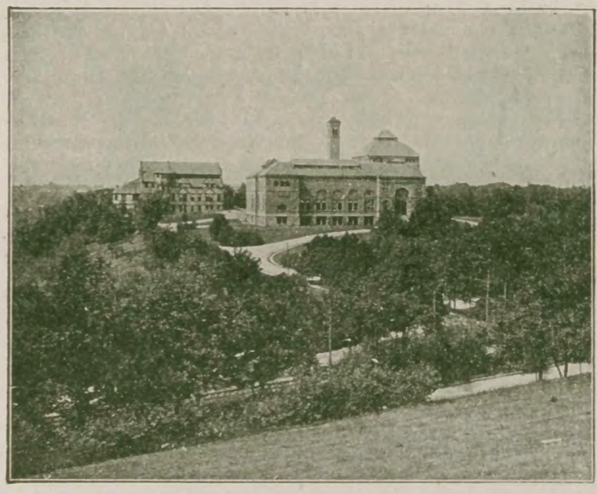

**Art Museum and Art School in Eden Park**

The Ohio Mechanics Institute, the oldest institution of its kind in the United States, is now occupying a new $600,000 home. The Institute has a rapidly-growing Industrial Museum. It maintains the only school of lithography in America. Such a school should be located here, as Cincinnati has some of the best-known lithographic establishments, each of which requires a large staff of well-trained artists. Cincinnati is one of the great theatrical poster printing centers of the country.

The public schools are educating the children in art. Courses in art are offered and all children have free access to the Art Museum when accompanied by their teachers. High School students desiring to make art their vocation can take a course which permits them to attend the Art Academy part of each day. By similar co-operation between the Art Academy and the Teachers' College of the University of Cincinnati, a teachers' art course is made possible. This course prepares advanced art students to supervise and teach art and the crafts in public and private schools. In addition, the Art Museum provides a course of lectures for teachers on the history of art.

The practical result of art training in Cincinnati is found in the efforts of the people to beautify their surroundings. There is a well-defined tendency to eliminate unsightly buildings, while the ever-growing park system speaks for itself.

### Hotels for All

Cincinnati has hotel accommodations of the highest character. Its two largest hotels, the Hotel Sinton and the Hotel Gibson, are diagonally across the street from each other and offer a combined room capacity of 2,000. Each has a splendid Convention Auditorium suitable for the largest convention sessions, with several smaller halls and meeting places. As many as two large and several small conventions have been conducted at the same time in a highly satisfactory manner at these two hotels. There are also other first-class hotels and a number of medium-priced houses readily accessible.

Besides spacious meeting places in the hotels, there are even larger accommodations, such as the Emery Auditorium in the Ohio Mechanics Institute Building, seating over 2200, and the Music Hall seating 3500.

Music Hall has two exhibit halls each 90 x 290, one with two floors, and the other with a balcony second floor. Both halls are connected through Music Hall, making the total floor space available, 97,549 feet. Halls are equipped with power and are lighted and heated. The Ohio National Guard Armory also has large space available for exhibitions.

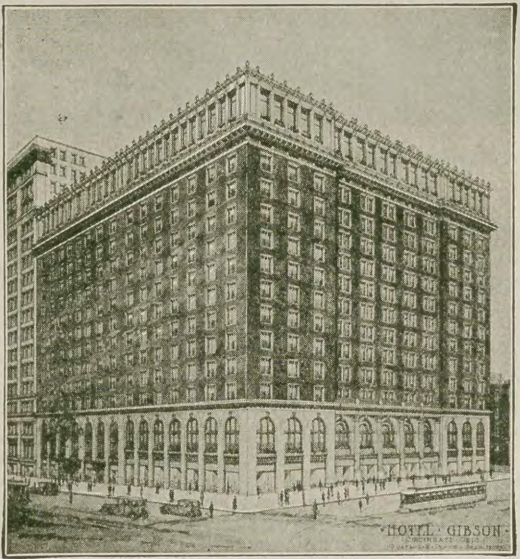

**Hotel Gibson**

A feature of the coming meeting will be the dedication of the Callahan Memorial which has been erected to the memory of the late Dr. J. R. Callahan, eminent dentist and dental research worker.

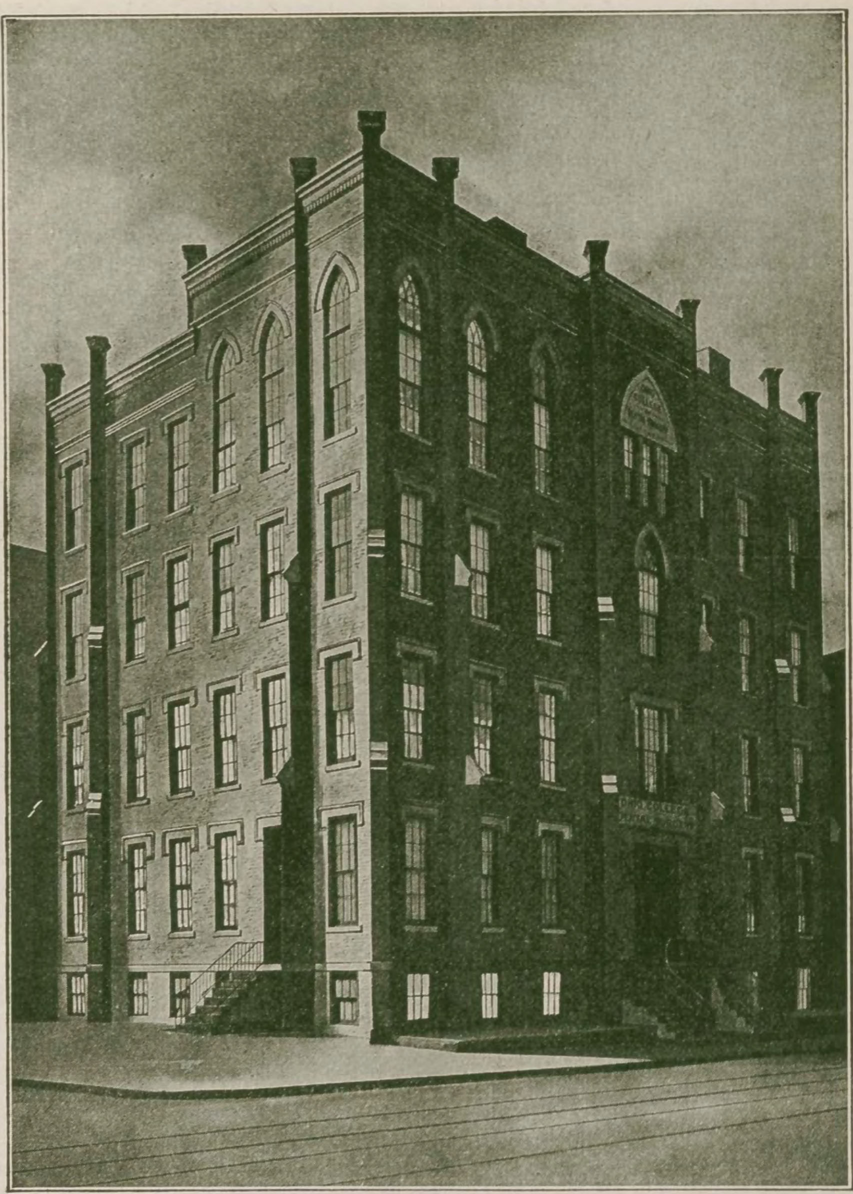

**Founded 1845**

**Building of the Ohio College of Dental Surgery, 7th and Mound Streets, Cincinnati, O.**

At the same time as the Ohio State Dental Society Meeting, the American Academy of Periodontology will hold its ninth annual meeting December 4 to 6, 1922.

The new section of the Hotel Gibson will be completed as shown in picture by the time the convention is to be held and will make additional accommodation for the visiting delegates to the Ohio State Dental Society Fifty-seventh Annual Meeting, December 5, 6 and 7, 1922.

Visiting delegates attending the Ohio State Dental Society meeting will remember that Cincinnati is a center for Dental Education which is ably carried forward by the two dental colleges, namely, The Ohio College of Dental Surgery and The Cincinnati College of Dental

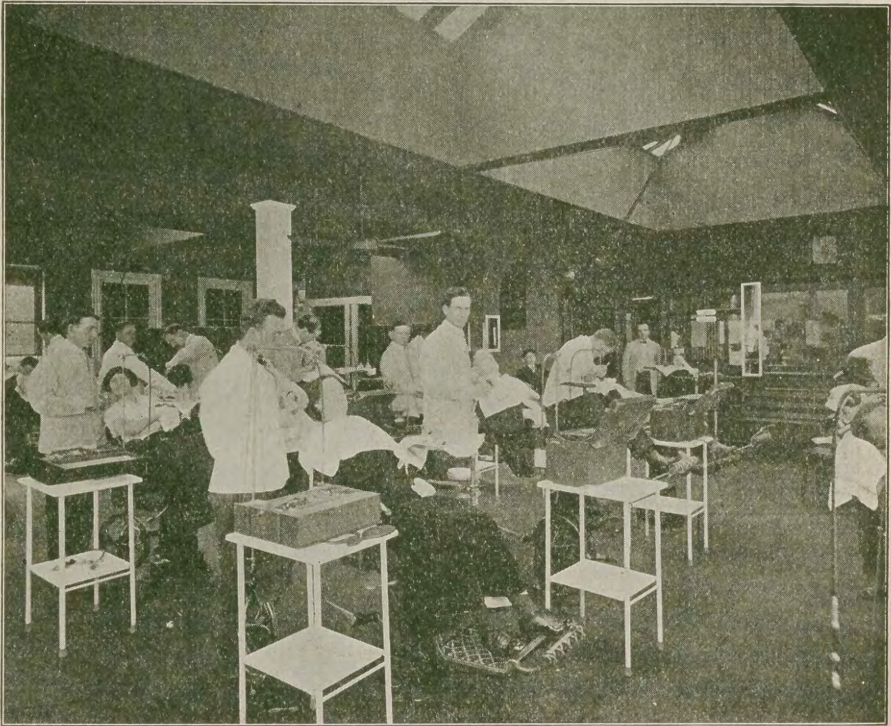

**View of the Operative Clinic of the Cincinnati College of Dental Surgery**

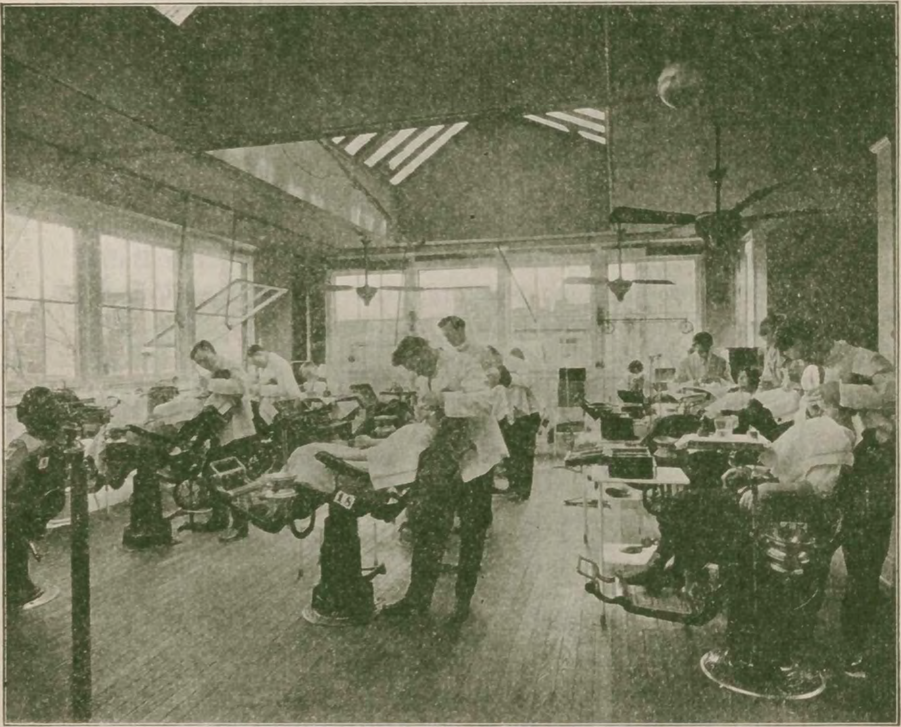

**Another View of the Operative Clinic of the Cincinnati College of Dental Surgery**

Surgery. From the halls of these colleges have come and are coming men proficient in the dental science. Cincinnati stands well to the front among American cities as a Dental Educational Center on account of the presence in her midst of these two great dental institutions.

The Cincinnati College of Dental Surgery offers complete courses leading to the degree D.D.S. Every facility is afforded the student in the study of this chosen profession; a comprehensive course of study; able professors and full equipment of dental apparatus. The college building is located on Court Street near Plum Street and contains 8,400 square feet of floor space. Large clinic in daily operation.

The Ohio College of Dental Surgery has completed three-quarters of a century of its existence. Its establishment in Cincinnati in 1845, largely through the efforts of its first president, was coincident and associated with the establishment of dentistry as a profession in this country. It became the pioneer of dentistry in all the territory west of the Alleghenies, and was the second college of dentistry established in the world. The Baltimore Dental College was first, the Ohio Dental College second. Early in its history the college erected its own building, which it occupied continuously for nearly half a century, sending out each year men who became pioneers and teachers of dentistry in this and other countries.

### PROGRAM

OF THE

### Ohio State Dental Society

FIFTY-SEVENTH ANNUAL SESSION

December 6-6-7, 1922

DEDICATION OF CALLAHAN MEMORIAL

Gibson Hotel Cincinnati, Ohio

---

### TUESDAY MORNING, DECEMBER 5th

**Manufacturers and Dealers Clinics**

Registration, Mezzanine Floor....................8:30
First General Session, Gibson Hotel Ball-Room....10:00
Meeting, Board of Directors, Ball-Room Floor,
right of elevator..........................11:00

### TUESDAY NOON

12:00 o'clock sharp

**Balanced Diet Luncheon**

Sidney J. Rauh, Chairman

Five minute talks on

Diet and Mouth Hygiene.

The Relation of Dentistry to General Social Problems and Public Health.

The Committee promises a novel program and some surprises.

---

### TUESDAY AFTERNOON

2:00 o'clock

"Partial Dentures"

By Charles A. Furrow................Tulsa, Okla.

Discussion by

I. Lester Furnas....................Cleveland, O.

"Preventive Dentistry"

By Arthur H. Merritt..............New York City

Election of Officers.

---

### TUESDAY EVENING

8:00 o'clock

"Mottled Enamel" (Original esearches)

By J. Leon Williams...................Solon, Me.

Discussion by

Arthur D. Black......................Chicago, Ill.

Weston A. Price......................Cleveland, O.

"The Principle of Diffusion Applied to the Callahan Method of Pulp Canal Filling"

"The Positive Destruction of Apical Infection by Electrolytic Medication"

By Harry B. Johnston................Atlanta, Ga.

Discussion by

R. Ottolengui.......................New York City

### WEDNESDAY MORNING

10:00 to 12:00 o'clock

Oral Surgery Clinics.......Cincinnati General Hospital

---

### WEDNESDAY AFTERNOON

1:30 o'clock

**Unveilling of Memorial to John R. Callahan**

Memorial Address

R. Ottolengui........................New York City

Presentation of Award to

J. Leon Williams, Solon, Me.

By Weston A. Price.................Cleveland, O.

---

### WEDNESDAY EVENING

6:00 o'clock sharp

**Informal Dinner Gibson Hotel Ball-Room**

In Honor of J. Leon Williams

---

### WEDNESDAY EVENING

8:00 o'clock

(Program of the Session furnished by The American Academy of Periodontology)

"The Bacteriology of Focal Infections"

By John C. Meisser...............Rochester, Minn.

"Blood Chemistry as a Diagnostic Aid in Focal Infections"

By R. N. DeNiord...................Buffalo, N. Y.

Discussions by

Martin H. Fischer...................Cincinnati, O.

John Oppie McCall...................Buffalo, N. Y.

### WEDNESDAY AND THURSDAY MORNING

### Lecture Clinics

Beginning at 9:00 o'clock

"Partial Dentures"
Charles Furrow........................Tulsa, Okla.

"Gold Inlay Technique"—Indirect Method
Toledo Clinic Club......................Toledo, O.

"Root Canal Technique"
Detroit Clinic Group.................Detroit, Mich.

"Cast Gold Inlays"
Roscoe H. Volland..................Iowa City, Ia.

"Elimination of Pus Pockets"
Arthur D. Black......................Chicago, Ill.
Frederick W. Merrifield...............Chicago, Ill.

"Operative Procedure for Removal of Cysts"
H. W. McMillan.....................Cincinnati, O.

"Orthodontia"
Wilson H. Foster....................Cincinnati, O.

"Surgical Removal of Mylo-hyoid Ridge"
W. B. Caldwell.......................Hamilton, O.

"Roentgen Ray as Guide in Pyorrhea"
R. R. Roush.........................Columbus, O.

"Removable Bridge, Wrought Clasps"
M. E. Neiswonger......................Dayton, O.

"Fixed Bridge"

"Porcelain Jacket Crown"
E. W. Graebner.......................Cleveland, O.

"Full Denture Construction"
Cincinnati Clinic Club...............Cincinnati, O.

"Radicgraphic Interpretation"
John Oppie McCall..................Buffalo, N. Y.

the latter said, that in his opinion the surest way to success as a fiction writer was to store one's mind with facts and you could then distort them at your leisure—which is all very well for avowed purposes of amusement, but the public health and public confidence are not matters to be legitimately trifled with and certainly not for commercial gain. It is but fair to grant that a proportion of the advertising claims for these cure-all dentifrices are based upon insufficient knowledge and with an honest intent to popularize an intrinsically good thing, but there is also manifest in a goodly proportion of dentifrice advertising the tactics of the quack and terror-monger, who prey upon the credulity and fears of the ignorant and superstitious. It is they whose dishonest practices and impossible claims should be suppressed in the interests of public health and public morals, for they constitute the modern exemplification of St. Mark's prophecy, that "false prophets shall rise and shall shew signs and wonders to seduce, if it were possible, even the elect."

The medical profession has for years waged effective warfare against these excrescences upon the fair name and reputation of their professional calling. It would seem that the time is full ripe for a dental crusade in the same direction.—*Dental Cosmos*, October, 1922.

---

### ANNOUNCEMENTS

STATE BOARD OF DENTAL EXAMINERS

The Michigan Board will meet November 13 to 18, 1922. The meeting will be held in the College of Dental Surgery.

---

THE OHIO STATE DENTAL SOCIETY

The Ohio State Dental Society will hold its annual meeting December 5, 6 and 7 in the Hotel Gibson of Cincinnati. A complete program will be published soon.

### AMERICAN ACADEMY OF PERIODONTOLOGY

The American Academy of Periodontology will hold its annual meeting in the Hotel Gibson, of Cincinnati, December 4 and 6, 1922.

---

### AMERICAN INSTITUTE OF DENTAL TEACHERS

The Thirtieth Annual Meeting of the American Institute of Dental Teachers will be held at Creighton University, Omaha, Neb., Hotel Fontenelle, headquarters, January 22, 23, 24 and 25, 1923.

A cordial invitation is extended to all persons interested in dental teaching. A. H. Hipple, President; Abram Hoffman, Secretary, 381 Linwood Ave., Buffalo, N. Y.

---

### INDEX OF DENTAL PERIODICAL LITERATURE

The Annual Index of Dental Periodical Literature for the year 1921 is now ready for delivery.

The contents have been prepared with the same care and attention to detail that has characterized the previous volumes and the make-up is similar in general to the books already published. This book contains all of the English periodical literature for the year and is complete to January 1, 1922. There are one hundred and forty-four $7\frac{1}{2} \times 10\frac{1}{2}$ inch pages securely bound with paper covers.

As the edition is limited to seven hundred and fifty copies, I suggest that everyone send his remittance by early mail.

Thanking you in anticipation, I beg to remain,

Yours very truly, A. Hoffman,

*Secretary-Treasurer.*

---

### INSTITUTE OF ORALOGY

The Institute of Oralogy requests us to print the following:

Out-of-town members of the medical and dental professions are extended the privilege of the Institute of

Oralogy offices, 587 Fifth Avenue, New York City, for consultation and examination of any of their visiting patients.

---

Colonel Herman A. Metz, president of the H. A. Metz Laboratories, Inc., was nominated for Congress on the Democratic ticket in the 17th Congressional District in the City of New York, in the primaries on September 20th.

Colonel Metz was formerly a representative in Congress from the 10th Congressional District of Brooklyn and made a very enviable record as a member of the lower House.

Colonel Metz was the first comptroller of the Greater City of New York, and through his efforts the city was placed on a firm financial foundation.

---

A press notice of the book, ''Modern Dentistry for the Laity,'' appearing in the magazine ''100% Management in the Executive's Bookshelf Department,'' September number, mentions the book as follows:

''Modern Dentistry for the Laity'' is the title of an informative little book by Alfred A. Crocker. Its subtitle reveals its significance for executives—''Industrial Dentistry for the Corporation, Modern Preventive Dentistry and Industrial Safety Welfare Dentistry.'' But in addition to industrial interest, the book contains many helpful tips for all who care for the health of their teeth. Published by THE DENTAL REGISTER, Cincinnati.

If you have not read this book it will be of interest to you to do so. Most of the libraries in the United States have a copy. If your library has not a copy, we wish to know, so we can send one.

THE SAM'L A. CROCKER CO.,
Cincinnati, O.

